# Latitudinal Variation in the Responses of Invasive *Alternanthera philoxeroides* and Native *Digitaria sanguinalis* to Soil Legacy Effects

**DOI:** 10.3390/plants15182792

**Published:** 2026-09-11

**Authors:** Hao Wu, Qianwen Yang, Hanfei Yang, Leilei Qiao, Benqiang Rao

**Affiliations:** 1 College of Life Sciences, Xinyang Normal University, Xinyang 464000, China; 2 Henan Dabieshan National Field Observation and Research Station of Forest Ecosystem, Zhengzhou 450046, China; 3 Xinyang Academy of Ecological Research, Xinyang 464000, China

**Keywords:** plant invasion, latitude, *Alternanthera philoxeroides*, soil legacy effects, ecological stoichiometry, photosynthetic fluorescence

## Abstract

Plant–soil interactions are increasingly recognized as a key driver of plant invasion, yet whether soil legacy effects associated with invaded habitats differentially influence invasive and native plants across broad geographic ranges remains poorly understood. We hypothesized that soil legacy effects would differ among soil sources from different latitudes and would affect the two species asymmetrically. Here, we address this gap by examining how latitudinal variations in soil legacy effects alter the growth and photosynthetic performance of both invasive *Alternanthera philoxeroides* and the co-occurring native *Digitaria sanguinalis*. We collected soil samples from 40 *A*. *philoxeroides*-invaded plots spanning 21° N to 37° N in China and pooled soils from five geographically adjacent sites at similar latitudes, generating eight composite soil sources (clusters 1–8, from low to high latitudes). We then conducted pot experiments with the soils originating from different latitudinal clusters to examine the effects of soil legacies on the morphology, biomass, nutrient content, and photosynthetic fluorescence of *A*. *philoxeroides* and *D*. *sanguinalis*. We found that soil legacy effects increased the maximum stem length and leaf area of *D*. *sanguinalis*, while they decreased the overall root–shoot ratio of plants at low and middle latitudes. Soil legacy effects altered the nitrogen–phosphorus ratio (N:P) of *A*. *philoxeroides* in most latitudinal clusters, with a pronounced shift toward greater phosphorus investment. In latitudinal cluster 8, soil legacy effects resulted in higher *F*_0_ and *F*_m_ values in *D*. *sanguinalis* than in *A*. *philoxeroides* under monoculture, indicating stronger PSII reaction center activity in the native species. In mixed culture, soil legacy effects eliminated the photosynthetic superiority of *A*. *philoxeroides* over *D*. *sanguinalis*. With increasing latitude, soil legacy effects shifted the photosynthetic fluorescence characteristics of the two studied species from being associated with plant growth to being associated with ecological stoichiometry. Our study indicates that soil legacy effects in *A*. *philoxeroides*-invaded habitats may not consistently promote *A*. *philoxeroides* invasion. Instead, their regulation of *A*. *philoxeroides* and *D*. *sanguinalis* performance is latitude-dependent. These findings provide a new perspective for understanding the geographic variation in mechanisms underlying plant invasion.

## 1. Introduction

Plant invasion significantly alters the community structure and interspecific relationships of native plants and concurrently modifies soil properties in invaded habitats, posing serious threats to global biodiversity, ecosystem functions, and agricultural production [1]. Invasive plants often outperform native species due to mechanisms such as enemy release, allelopathy, and more efficient resource-use strategies [2]. During the invasion process, these plants restructure native community composition through resource competition and interspecific interactions [3] while concurrently modifying soil physicochemical properties and microbial communities via root exudates and litter decomposition [4,5]. Furthermore, ongoing global changes are altering the distribution and ecological consequences of invasive plants [6]. For instance, climate warming increases the thermal suitability of high-latitude regions, allowing many invasive plants to expand into previously unsuitable areas where establishment was constrained by low temperatures [7,8]. Meanwhile, increasing atmospheric nitrogen deposition alters soil nitrogen availability and nutrient limitation regimes [9]. Increased nitrogen inputs may alleviate nitrogen limitation and enhance soil resource availability. This could confer a competitive advantage to invasive plants, which typically exhibit high nutrient demand and efficient resource acquisition [10]. Therefore, investigating plant–soil interactions in invaded ecosystems across larger geographic scales can help identify the environmental drivers of invasion and facilitate the development of cross-regional collaborative prevention and control measures.

Soil legacy effects refer to the phenomenon whereby invasive plants alter soil physicochemical properties and biotic communities, persisting after invader removal and exerting lasting effects on subsequent plant growth, including that of native and other invasive species [11]. Due to the diversity of species traits and environmental conditions, the direction and intensity of soil legacy effects vary across habitats [12]. During plant invasion, soil legacies are not determined solely by the invasive plant species; large-scale habitat heterogeneity also modifies soil physicochemical characteristics and microbial community composition, which can either promote or inhibit plant invasion [5]. If invasive plants benefit more from soil legacies than native plants, these legacies can act as a crucial driver of plant invasion [13]. For example, under nitrogen deposition, soil legacies exert a stronger facilitative effect on the invasive plant *Rhus typhina*, whereas their effect on the native *Ailanthus altissima* shifts to inhibition [14]. Similarly, the invasive *Ageratina adenophora* can aggregate beneficial soil bacteria through root exudates, which increase soil ammonium nitrogen and available phosphorus contents and alleviate autotoxicity, thus accelerating its invasive expansion [15]. In addition, the accumulation of local pathogens hypothesis (ALPH) suggests that invasive plants gradually accumulate local soil pathogens in invaded habitats and that these pathogens are more harmful to native plants than to invasive ones, thereby conferring a competitive advantage to the invaders [16]. However, some studies have reported that the soil legacies of invasive plants are not always positive. As invasion progresses, invasive plants gradually accumulate obligate pathogens and experience soil biotic suppression, leading to reductions in survival rates, biomass, and population sizes and ultimately resulting in negative feedback on plant invasion [17]. In summary, soil legacy effects vary in direction and intensity depending on habitat conditions. Nevertheless, how soil legacy effects of invaded habitats vary across broad geographic ranges in their impacts on invasive and co-occurring native plants remains unclear.

Latitudinal variations shape diverse habitats by regulating temperature and precipitation regimes, which in turn affect the performance of invasive and native plants, as well as their coexistence patterns [18]. Studies have demonstrated that the morphological plasticity and ecological adaptability of invasive plants increase with latitude and that these processes strongly suppress native plant communities at relatively high latitudes [3,9]. In high-latitude regions, invasive plants experience lower insect feeding pressure than their native counterparts, and this natural enemy release effect facilitates plant invasion at higher latitudes [19]. Soil physicochemical properties are fundamental to soil function and are strongly influenced by latitudinal gradients [20]. Soil organic carbon, organic matter, and humus contents rise with latitude, whereas litter decomposition rates decline. Moreover, freeze–thaw cycles in high-latitude regions can suppress plant growth [21]. At higher latitudes, plant invasion enhances soil enzyme activity, accelerating organic matter decomposition and mineralization, which improves soil nutrient availability and facilitates invasion success [22]. Additionally, Cheng et al. reported that native plants exhibited a shift from negative to positive responses to invaded soils with increasing latitude, whereas invasive plants maintained positive responses across different latitudes [18]. This asymmetric latitudinal trend in soil legacy effect may facilitate invasion success across a wide range of latitudes. As abiotic stress intensifies at higher latitudes, changes in soil properties along the latitudinal gradient become the dominant factor affecting plant invasion [23].

Native to South America, *Alternanthera philoxeroides* has now invaded more than 20 provinces across China because of its strong ecological adaptability, asexual reproduction, and amphibious growth. This invasive species causes severe damage to native plant diversity and ecological environments and is listed on China’s List of Key Managed Alien Invasive Species [24]. Some studies have revealed a coupled relationship between *A*. *philoxeroides* invasion and soil properties. For example, *A*. *philoxeroides* invasion alters soil chemical properties, and the soil nutrient content increases with invasion severity [25]. Allelopathic substances secreted by *A*. *philoxeroides* roots inhibit the growth of native plants by reducing soil microbial metabolic activity, resulting in an imbalance in soil microbial community structure [26,27]. *A*. *philoxeroides* significantly promotes its own aboveground biomass accumulation and enhances its resistance to soil pathogens through symbiosis with arbuscular mycorrhizal fungi in invaded areas [28]. Furthermore, an increase in latitude enhances the invasive dominance of *A*. *philoxeroides* in natural communities, whereas global warming increases its interspecific competitiveness and accelerates its spread to higher latitudes [1,7]. The strength of interspecific association and the proportion of positively/negatively associated species pairs in terrestrial *A*. *philoxeroides*-invaded communities decrease with increasing latitude, suggesting that *A*. *philoxeroides* invasion reshapes the interspecific relationship network of communities and that its relationship with native plants also varies with latitude [24]. *Digitaria sanguinalis*, a native annual herb with strong tillering ability and a relatively tall plant type, co-occurs extensively with terrestrial *A*. *philoxeroides* in natural habitats [7,8,24]. Wu et al. found that *D*. *sanguinalis* had the highest importance value (IV) among accompanying plant species in terrestrial *A*. *philoxeroides*-invaded communities across the 21° N–37° N latitudinal range, and the co-occurring IV ratio of *A*. *philoxeroides* to *D*. *sanguinalis* was approximately 6:1 [24,29]. Although previous studies have investigated interspecific competition between *A*. *philoxeroides* and *D*. *sanguinalis* [8], the effects of latitudinal variation in soil legacies on the performance of both species remain unclear, hindering our understanding of the latitudinal patterns of *A*. *philoxeroides* invasion.

Currently, geographic variations in plant–soil interactions have received increasing attention; however, studies on the soil legacy effects of large-scale field-sourced soils for terrestrial invasive herbaceous plants are lacking. Existing research has largely been confined to single response metrics, lacking systematic integration of plant growth, nutrient stoichiometry, and photosynthetic physiology. Furthermore, whether soils from invaded habitats of different latitudinal origins exert asymmetric effects on invasive versus native plants remains unclear. In this study, using field soil sampling and pot experiments, we investigated how soil legacy effects differ among soils from habitats invaded by *A*. *philoxeroides* at different latitudes and how these differences affect the performance of the native species *D*. *sanguinalis*. We proposed three testable hypotheses: (1) Soils collected from low-latitude invaded habitats generate growth-promoting effects, while high-latitude soils weaken the invasion dominance of *A*. *philoxeroides*. (2) Soil legacy effects induce divergent nitrogen and phosphorus acquisition strategies in *A*. *philoxeroides* and *D*. *sanguinalis* across different latitudes. (3) As latitude increases, dominant regulators of plant photosynthetic fluorescence shift from growth-related indicators to nutrient stoichiometry traits. Specifically, we addressed the following questions: (1) Do soil legacy effects at different latitudes differentially influence the growth traits (morphology, biomass, and nutrient) and photosynthetic fluorescence of *A*. *philoxeroides* and *D*. *sanguinalis*? (2) Do soil legacy effects at different latitudes alter the plant growth traits most closely associated with photosynthetic fluorescence?

## 2. Results

### 2.1. Effects of Soil Legacy on Plant Morphology

**Figure 1:**
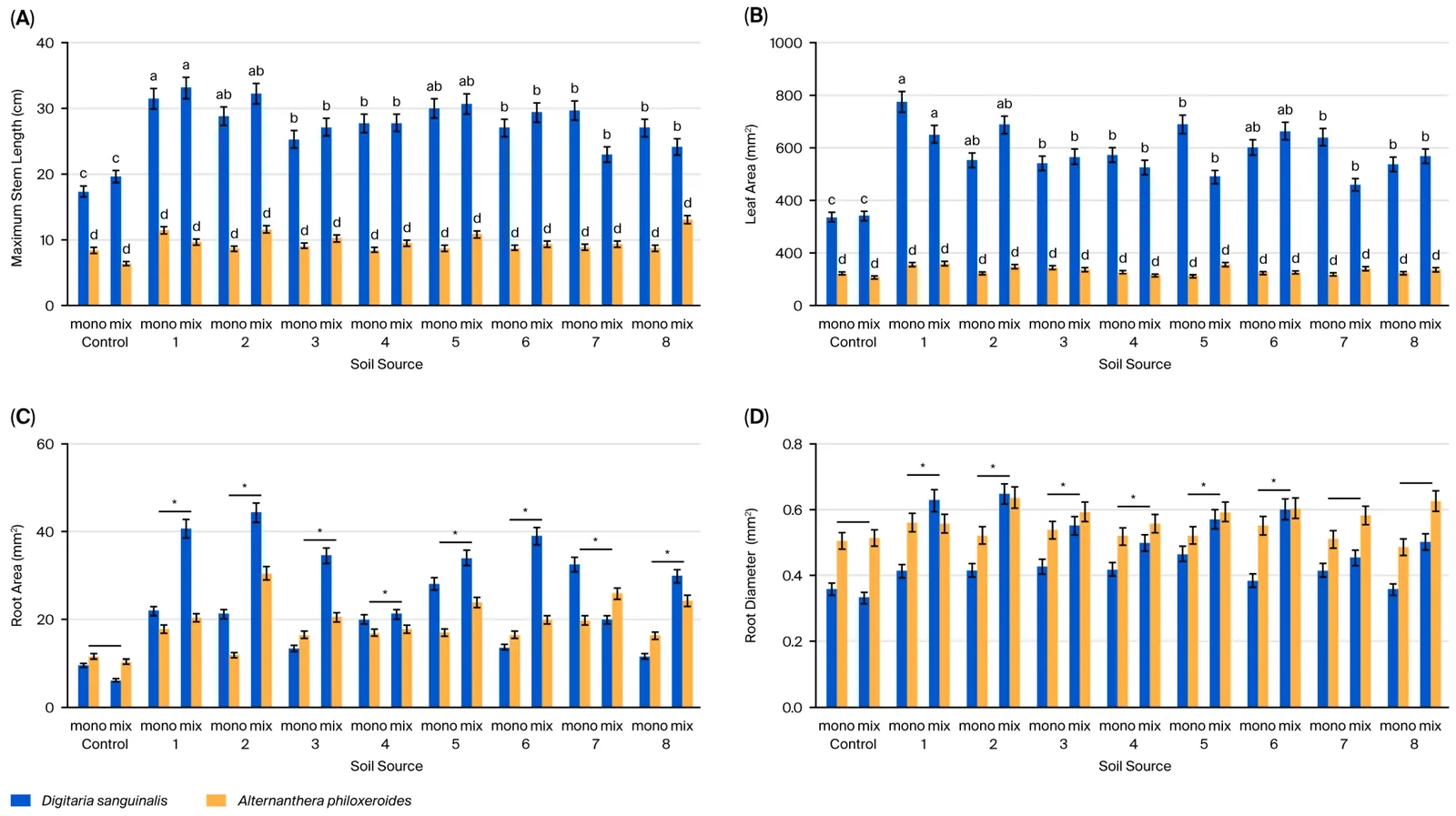
Multiple comparisons of (**A**) maximum stem length, (**B**) leaf area, (**C**) root area, and (**D**) root diameter among treatments. **Abbreviations:** mono—monoculture; mix—mixed culture. Arabic numerals 1–8 represent the soil legacies from latitudinal clusters 1 to 8. Different lowercase letters indicate significant two-way interaction effects between soil source and species identity (*p* < 0.05), and asterisks denote significant differences from the control treatment within each corresponding soil legacy (*p* < 0.05).

The interaction between soil source and species attribute significantly affected the maximum stem length and leaf area, whereas the single effect of soil treatment significantly affected the root area and root diameter (Table 1). Multiple comparisons revealed that, compared with the control treatment, soil legacies from all latitudinal clusters significantly increased the mean stem length and leaf area of *D*. *sanguinalis*, with the largest increases observed in cluster 1, where these traits increased by 74.759% and 110.600%, respectively (Figure 1A,B). In contrast, these traits in *A*. *philoxeroides* were not significantly affected by soil legacies (Figure 1A,B). Compared with the control treatment, soil legacies significantly increased the root area in all latitudinal clusters (Figure 1C) and significantly increased the root diameter at low and middle latitudes (Figure 1D).

### 2.2. Effects of Soil Legacy on Plant Biomass

**Figure 2:**
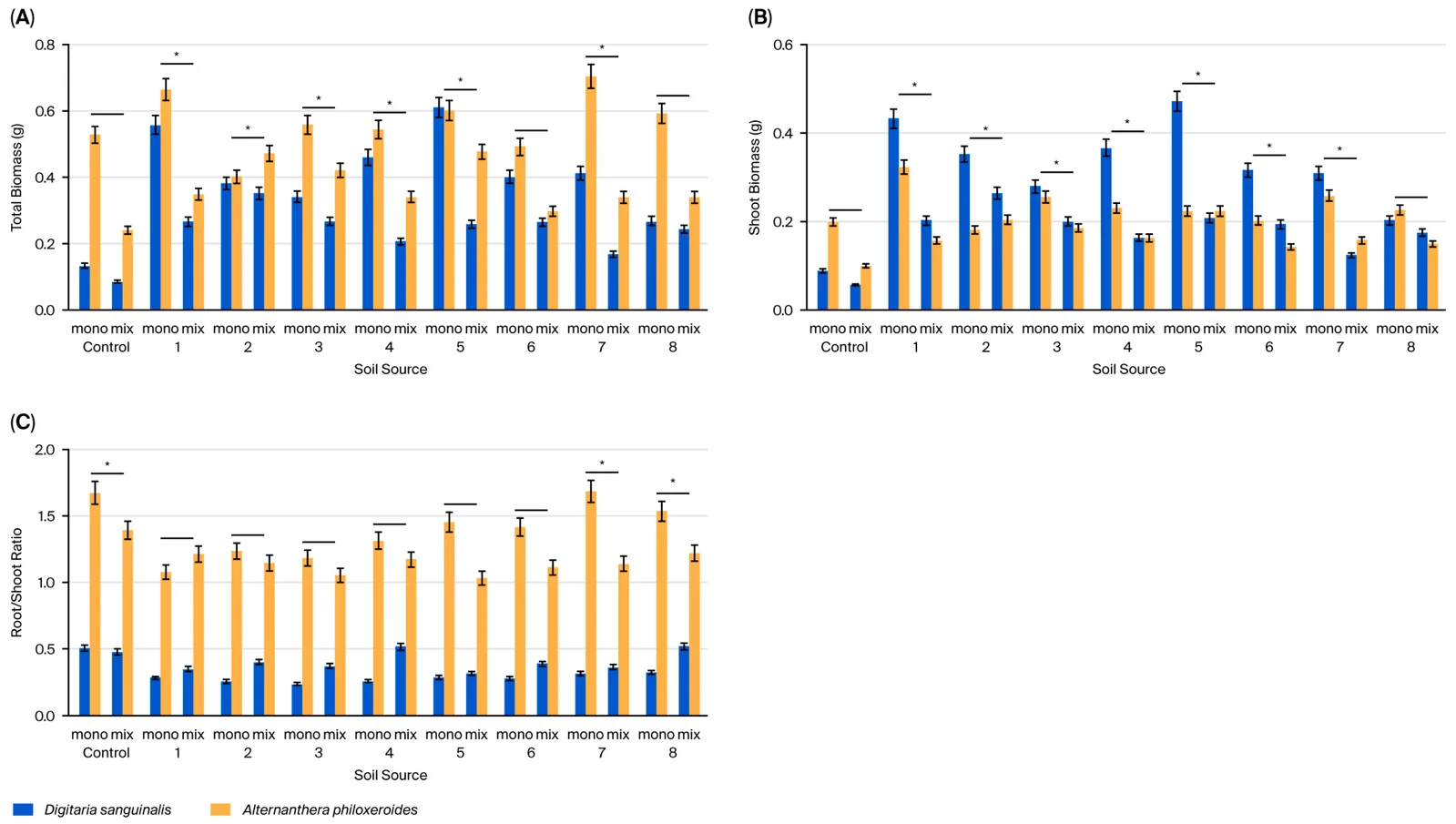
Multiple comparisons of (**A**) total biomass, (**B**) shoot biomass, and (**C**) the root–shoot ratio among treatments. Arabic numerals 1–8 represent the soil legacies from latitudinal clusters 1 to 8. Asterisks denote significant differences from the control treatment within each corresponding soil legacy (*p* < 0.05).

The single effect of soil source significantly affected the total biomass, shoot biomass, and root–shoot ratio (Table 2). Multiple comparisons revealed that, compared with the control treatment, soil legacies significantly increased the total biomass in all latitudinal clusters, except for clusters 6 and 8, with the largest increases observed in clusters 1 and 5, where the total biomass increased by 86.404% and 97.606%, respectively (Figure 2A). Except for cluster 8, soil legacies significantly increased shoot biomass in all other clusters, with the largest increases observed in clusters 1 and 5, where shoot biomass was 149.978% and 152.747% greater than that of the control, respectively (Figure 2B). Soil legacies significantly decreased the root–shoot ratio at low and middle latitudes (Figure 2C).

### 2.3. Effects of Soil Legacy on Plant Nutrient Content

**Figure 3:**
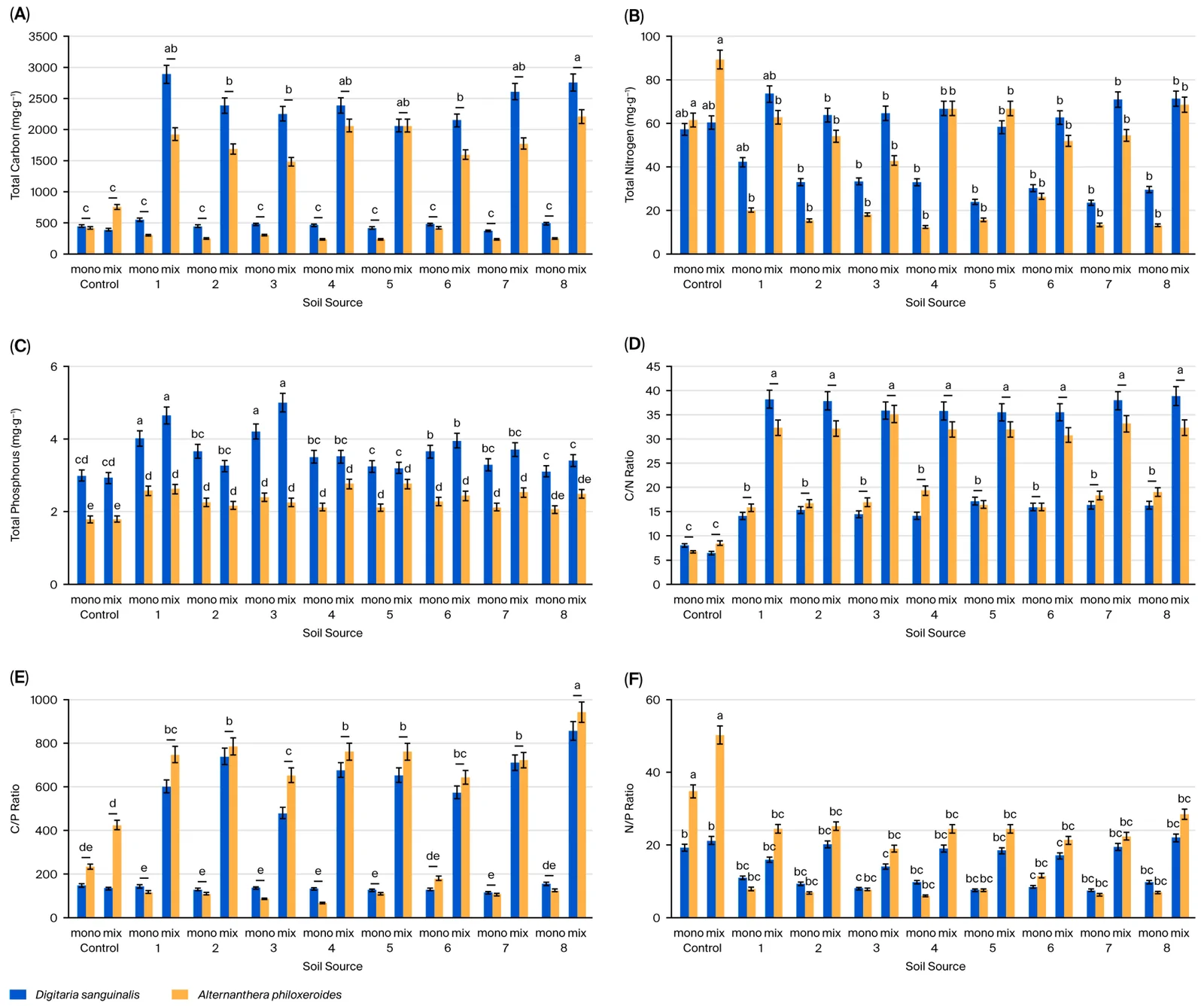
Multiple comparisons of (**A**) total carbon, (**B**) total nitrogen, (**C**) total phosphorus, (**D**) C:N, (**E**) C:P, and (**F**) N:P among treatments. Arabic numerals 1–8 represent the soil legacies from latitudinal clusters 1 to 8. In panels (**A**,**D**,**E**), different lowercase letters above the aggregation line indicate significant two-way interaction effects between soil source and planting type (*p* < 0.05). In panels (**B**,**C**,**F**), different lowercase letters indicate significant two-way interaction effects between soil source and species identity (*p* < 0.05).

The interaction between soil source and planting type significantly affected total C, C:N, and the C:P ratio, whereas the interaction between soil source and species identity significantly affected total N, total P, and the N:P ratio (Table 3). Multiple comparisons revealed that soil legacies from all latitudinal clusters significantly increased the total C, C:N, and C:P ratios in mixed culture, with the largest increases observed in cluster 8, where these traits increased by 333.415%, 376.241%, and 219.669%, respectively (Figure 3A,D,E). Soil legacies from all latitudinal clusters significantly reduced the total N of *A*. *philoxeroides* (Figure 3B), whereas soil legacies at clusters 1, 3, and 6 significantly increased the total P of *D*. *sanguinalis* by 46.458%, 38.051%, and 18.384%, respectively (Figure 3C). Soil legacies from most latitudinal clusters (except cluster 8) significantly increased total P in *A*. *philoxeroides* (Figure 3C), whereas those from all latitudinal clusters significantly reduced the N:P ratio in *A*. *philoxeroides* (Figure 3F).

### 2.4. Effects of Soil Legacy on Plant Photosynthetic Fluorescence

**Figure 4:**
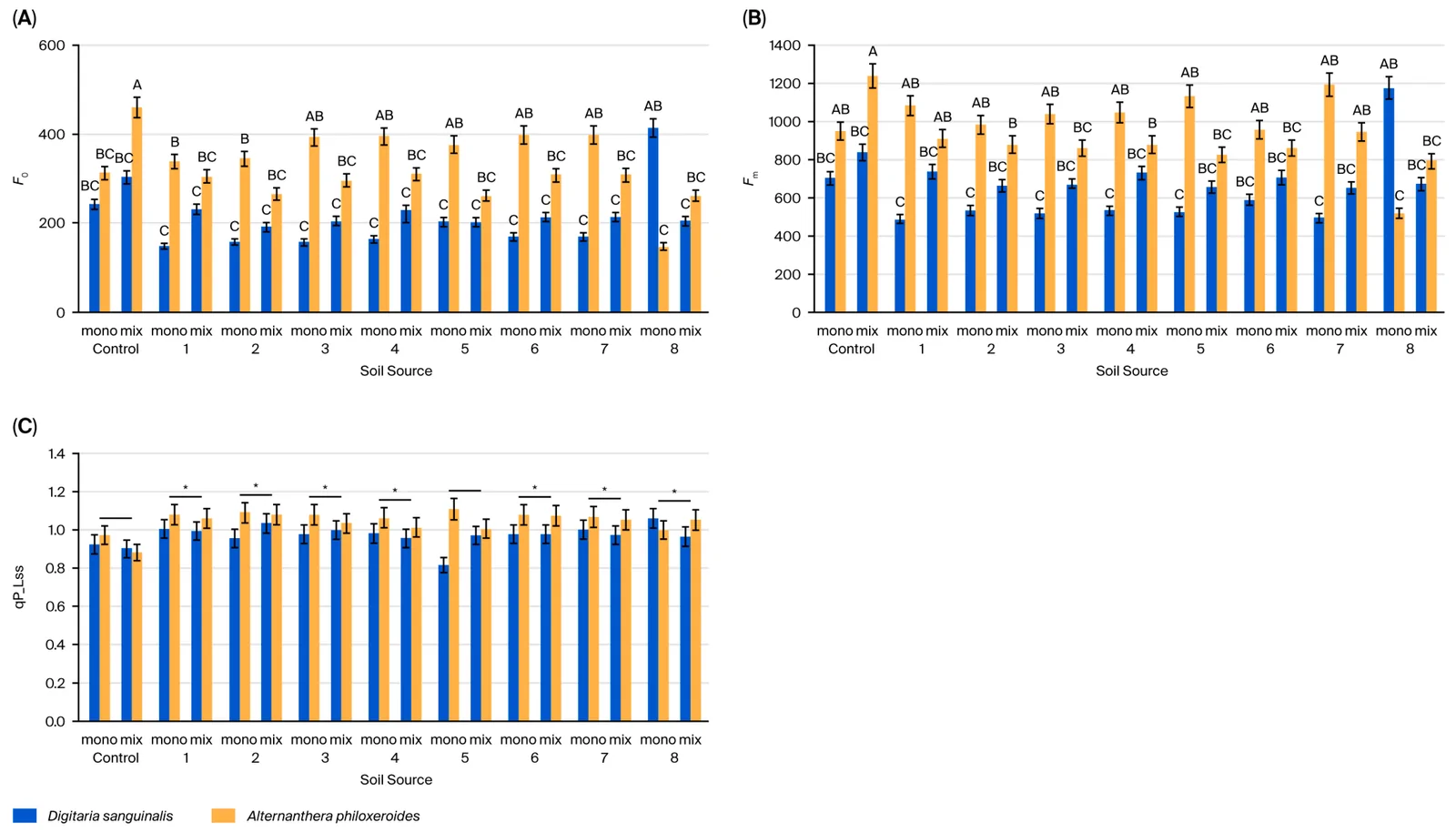
Multiple comparisons of (**A**) *F*_0_, (**B**) *F*_m_, and (**C**) qP_Lss among treatments. Arabic numerals 1–8 represent the soil legacies from latitudinal clusters 1 to 8. Different capital letters indicate significant three-way interaction effects between soil source, planting type, and species identity (*p* < 0.05), and asterisks denote significant differences from the control treatment within each corresponding soil legacy (*p* < 0.05).

The interactions among soil source, planting type, and species identity significantly affected *F*_0_ and *F*_m_. The single effect of soil source significantly affected qP_Lss (Table 4). Multiple comparisons revealed that, in the monoculture, soil legacies from clusters 1 to 7 resulted in significantly higher *F*_0_ in *A*. *philoxeroides* than in *D*. *sanguinalis*. In contrast, in cluster 8, soil legacies resulted in 181.892% higher *F*_0_ in *D*. *sanguinalis* than in *A*. *philoxeroides* in monoculture (Figure 4A). For latitudinal clusters 1 to 5 and 7, soil legacies resulted in significantly higher *F*_m_ in *A*. *philoxeroides* than in *D*. *sanguinalis* in monoculture. In cluster 8, however, the soil legacies resulted in 127.888% higher *F*_m_ in *D*. *sanguinalis* than in *A*. *philoxeroides* in monoculture (Figure 4B). Furthermore, soil legacies from all latitudinal clusters eliminated the significant advantages of *A. philoxeroides* over *D. sanguinalis* with respect to *F*_0_ and *F*_m_ in mixed culture (Figure 4A,B). Except for cluster 5, soil legacies significantly increased qP_Lss in all other latitudinal clusters compared with the control treatment (Figure 4C).

### 2.5. Effects of Soil Legacies on Growth–Photosynthetic Fluorescence Relationships Across Latitudes

**Figure 5:**
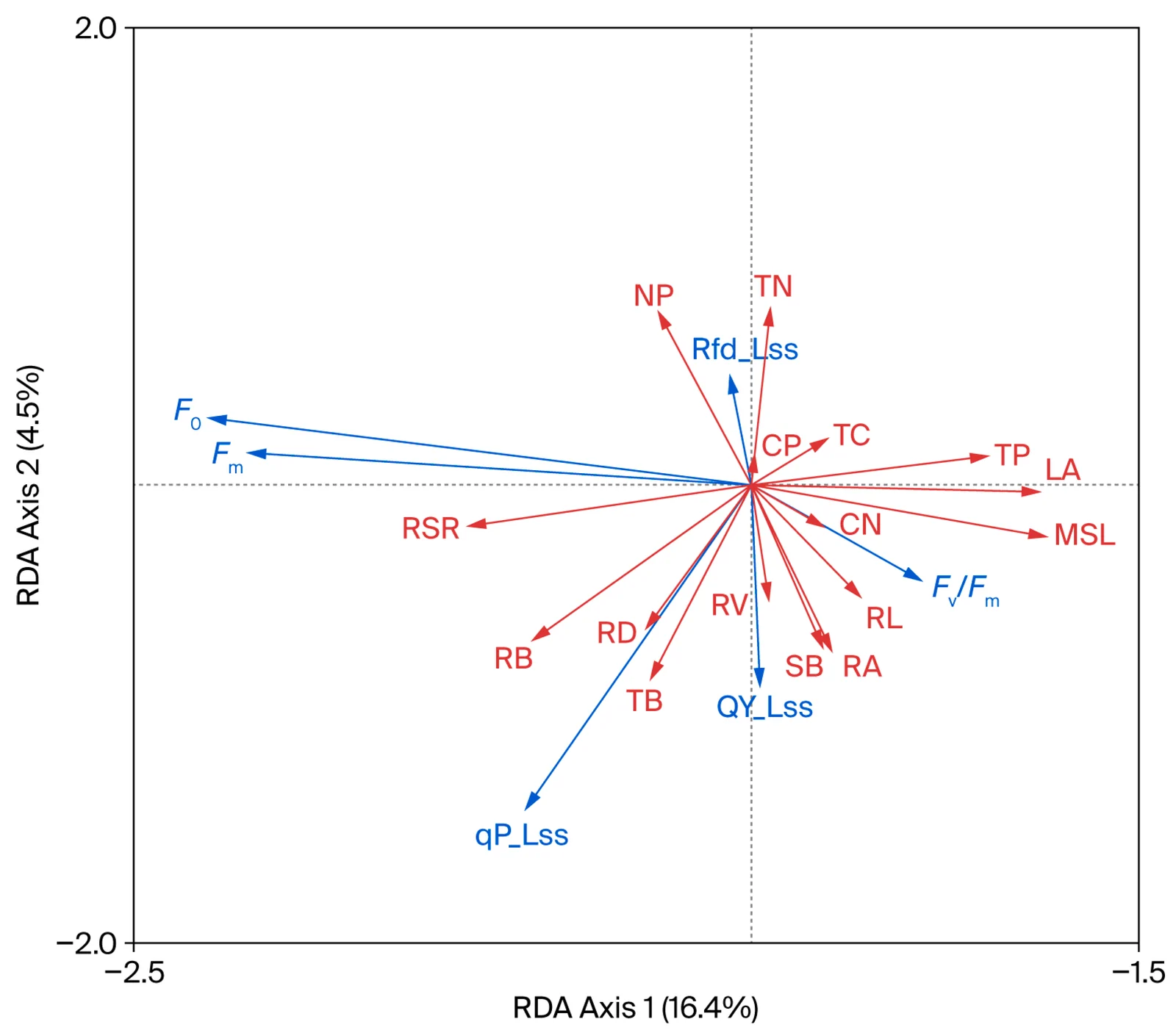
pRDA ordination diagram of growth and photosynthetic fluorescence indicators at the overall plant level. **Abbreviations:** *F*_0_—minimum fluorescence; *F*_m_—maximum fluorescence; *F*_v_/*F*_m_—maximum photochemical efficiency; qP_Lss—photochemical quenching; Rfd_Lss—steady-state fluorescence decay rate; QY_Lss—steady-state actual photochemical efficiency; LA—leaf area; RL—root length; RA—root area; RD—root diameter; RV—root volume; MSL—maximum stem length; TB—total biomass; SB—shoot biomass; RB—root biomass; RSR—root–shoot ratio; TC—total carbon; TN—total nitrogen; TP—total phosphorus; CN—carbon–nitrogen ratio (C:N); CP—carbon–phosphorus ratio (C:P); and NP—nitrogen–phosphorus ratio (N:P). Red vectors represent growth indicators, and blue vectors represent photosynthetic fluorescence indicators. The arrows indicate the direction of increasing values for each variable from the RDA ordination center. The angle between each pair of vectors indicates their correlation; the smaller the angle, the greater the correlation. The same abbreviations and vector interpretation apply to Figure 6.

Partial redundancy analysis (pRDA) revealed significant associations between certain growth indicators and the photosynthetic fluorescence indicators at the whole-plant level (Figure 5). Forward selection identified that the plant growth indicators with the highest individual contribution rates in the pRDA ordination model were total phosphorus (TP: 27.73%), leaf area (LA: 19.62%), total nitrogen (TN: 11.78%), and maximum stem length (MSL: 6.99%). These four indicators collectively showed significant marginal effects (all *p* < 0.05; see Table S1 for detailed adjusted *R*^2^ and *F*-statistics). In terms of directional relationships, the rapid fluorescence kinetics parameters *F*_0_ and *F*_m_ exhibited negative associations with TP, LA, and MSL, whereas *F*_v_/*F*_m_ exhibited the opposite trend for these three growth indicators (Figure 5). For steady-state parameters, qP_Lss and QY_Lss were negatively associated with TN, while Rfd_Lss was positively associated with TN (Figure 5).

**Figure 6:**
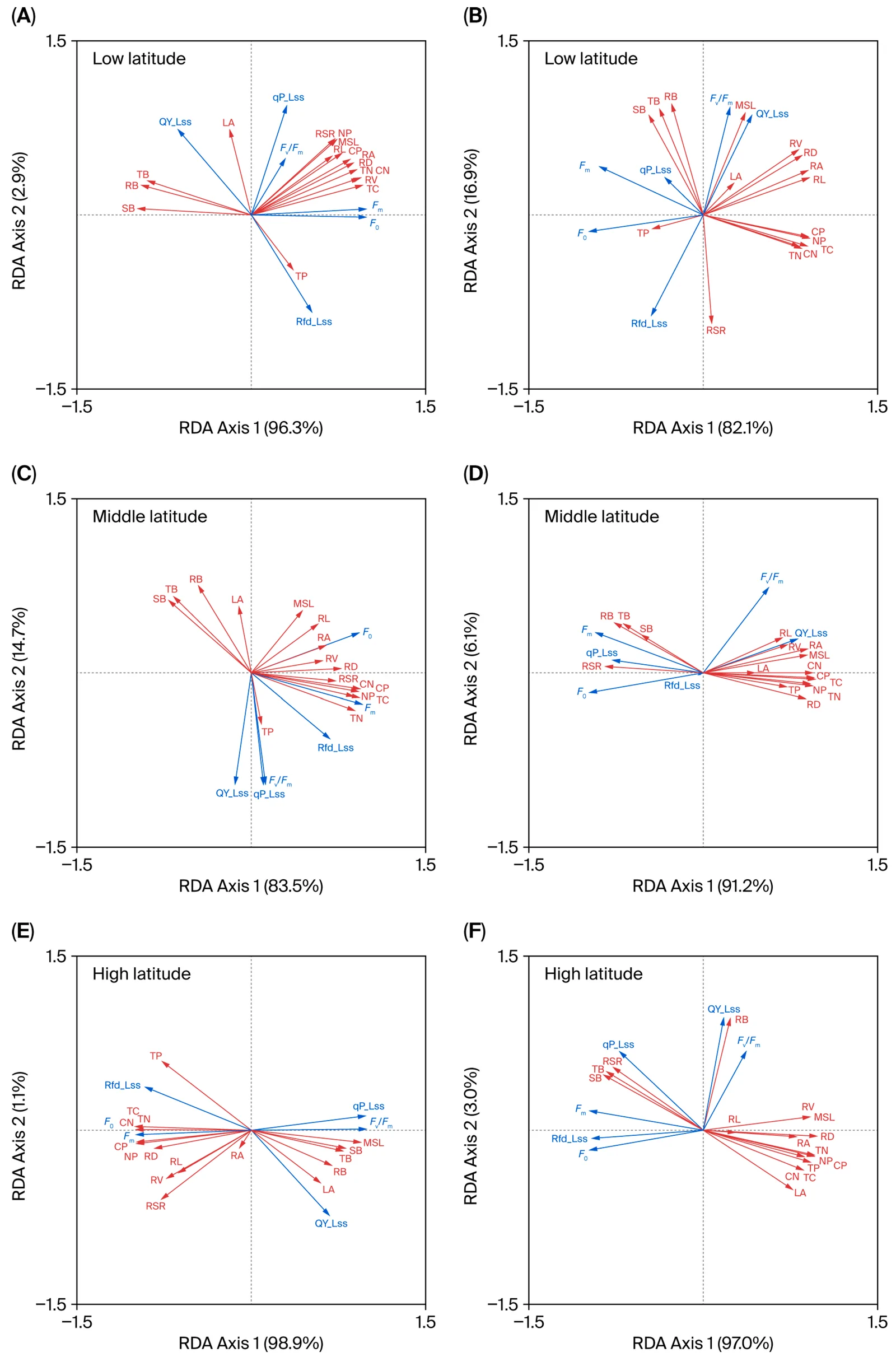
RDA ordination diagram of growth and photosynthetic fluorescence indicators under soil legacy effects at different latitudes. Panels (**A**,**C**,**E**) represent *D*. *sanguinalis* under low-, middle-, and high-latitude soil legacy effects, respectively. Panels (**B**,**D**,**F**) represent *A*. *philoxeroides* under low-, middle-, and high-latitude soil legacy effects, respectively.

Redundancy analysis (RDA) revealed that the first axis explained more than 80% of the cumulative variance in the coupling between plant growth and photosynthetic fluorescence across latitudinal clusters (Figure 6). For the low-latitude soil legacies (Figure 6A,B; Table S2), the dominant predictors for *D*. *sanguinalis* were shoot biomass (SB) and total carbon (TC), with *F*_0_ and *F*_m_ positively associated with TC but negatively associated with SB. In contrast, for *A*. *philoxeroides*, the dominant predictors were the carbon–phosphorus ratio (C:P), root length (RL), and nitrogen–phosphorus ratio (N:P), all of which showed negative associations with *F*_0_ and *F*_m_. In the middle-latitude soil legacies (Figure 6C,D; Table S2), the photosynthetic fluorescence of *D*. *sanguinalis* was strongly linked to the carbon–nitrogen ratio (C:N), TC, and C:P, with *F*_0_, *F*_m_, and Rfd_Lss all positively associated with these predictors. For *A*. *philoxeroides*, however, C:P, TC, and N:P emerged as the dominant predictors, all exhibiting negative associations with *F*_0_, *F*_m_, and Rfd_Lss. In the high-latitude soil legacies (Figure 6E,F; Table S2), the photosynthetic fluorescence of *D*. *sanguinalis* was associated with five dominant predictors—total nitrogen (TN), TC, C:N, N:P, and C:P, which all showed positive relationships with *F*_0_, *F*_m_, and Rfd_Lss. For *A*. *philoxeroides*, the dominant predictors shifted to root diameter (RD), N:P, C:P, and TN, all of which were negatively associated with *F*_0_, *F*_m_, and Rfd_Lss.

## 3. Discussion

### 3.1. Effects of Soil Legacy on Plant Growth Across Latitudes and Related Mechanisms

Invasive plants can increase their invasion success through root-mediated allelopathy or by recruiting soil microbial communities that benefit their growth [30]. However, in our study, soil legacies significantly increased the maximum stem length and leaf area in the native species *D*. *sanguinalis* but did not significantly affect these traits in the invasive species *A*. *philoxeroides*. This may be because high-density invasion zones of *A*. *philoxeroides* accumulate host-specific pathogens and root-knot nematodes in the rhizosphere, which suppress its further spread [31], whereas native species that are distantly related to *A*. *philoxeroides* may escape these negative soil-borne effects and respond positively to the soil properties altered by the invader [5]. Soil legacies may also improve soil nutrient conditions and introduce key functional microbes, which could contribute to the positive growth response of *D*. *sanguinalis*—an effect that is particularly evident in low-latitude regions where soil nutrient levels and enzyme activity are relatively high [32,33]. Our study revealed that soil legacies increased the root area across all latitudes while also enhancing root diameter at low and middle latitudes. This may be explained by the higher labile nutrient availability and microbial diversity in these regions, which promote root development. Under resource-rich conditions, plants prioritize the construction of thicker roots with greater transport efficiency than the expansion of the root system [34]. In contrast, high-latitude regions are constrained by low temperatures and reduced soil nutrient availability, leading plants to prioritize investment in fine roots over root thickening to maximize soil exploration. Consequently, soil legacies at high latitudes increase root surface area but do not significantly affect root diameter [35].

In this study, soil legacies increased total biomass and shoot biomass while reducing the root–shoot ratio. This pattern may reflect improved plant performance under certain soil legacy conditions, an effect accompanied by greater biomass accumulation and altered biomass allocation [36]. Consistent with this mechanism, compared with the control soils, soils conditioned by *A*. *philoxeroides* were richer in organic matter and mineral nutrients, with greater microbial activity that likely contributed to the observed growth promotion [17,37]. Furthermore, low-latitude regions, characterized by warm and humid climates, high biodiversity, and intense biogeochemical cycles, develop soils with high available mineral nutrients and strong microbial activity, thus providing stronger support for plant growth [38,39]. In contrast, extreme climatic conditions at higher latitudes limit soil microbial activity and nutrient availability [40], resulting in no significant effects of soil legacies on plant biomass in the high-latitude cluster (cluster 8) in our study. This observation aligns with Optimal Partitioning Theory, which posits that plants dynamically adjust their biomass allocation on the basis of resource availability [41,42]. Accordingly, in low- and middle-latitude areas with favorable water and heat conditions, plants tend to allocate more resources to aboveground parts to increase their photosynthetic efficiency, whereas in high-latitude regions where resources are scarce, plants invest more in belowground structures to improve their resource acquisition capacity [43].

Carbon, nitrogen, and phosphorus are essential elements for plant growth and metabolism, and their stoichiometric ratios reflect the efficiency of resource utilization and ecological adaptation strategies [44]. In this study, soil legacies increased total C, C:N, and C:P in the plant tissues under mixed culture. This is because carbon serves as a fundamental resource for tissue construction and the synthesis of defensive compounds, and its fixation efficiency directly influences competitive success [45]. A relatively high C:N ratio enhances the ability of plants to store resources and defend against stressors, whereas a relatively high C:P ratio indicates efficient nutrient resorption and the ability to maintain stable growth under stressful conditions [46]. Previous studies have shown that invasive species such as *Eupatorium adenophorum* and *Solidago canadensis* enhance competitive advantages by improving carbon assimilation and optimizing photosynthetic resource allocation; our results were consistent with these findings [47,48]. Notably, relatively low levels of active soil nutrients in high-latitude regions may stimulate plants to increase tissue carbon fixation rates to improve interspecific competitiveness [49]; accordingly, soil legacies in cluster 8 led to the greatest increases in tissue C concentration and C:P. Compared with native species, invasive plants typically exhibit higher nitrogen content [50], but in this study, soil legacies reduced total N in *A*. *philoxeroides*, suggesting that soil conditioning from invaded habitats did not further amplify its N acquisition advantage; instead, this shift prompted *A*. *philoxeroides* to allocate more resources toward phosphorus uptake. This may be due to invasive plants altering the soil microbial community structure through root exudates, thus increasing soil phosphorus activation and availability, which in turn reduces nitrogen availability and triggers adjustments in nutrient allocation strategies [26].

This shift in nitrogen and phosphorus allocation strategies was further reflected in the plant N:P ratios. When the N:P ratio exceeds 16, plant growth is primarily limited by phosphorus [51]. In this study, the N:P ratio of *A*. *philoxeroides* decreased under soil legacy effects, suggesting that soil legacies alleviated the phosphorus constraint on its growth and may have facilitated its invasion. Moreover, phosphorus limitation is prevalent in most terrestrial ecosystems in China [52,53]. Soil legacies can influence plant nutrient acquisition by modulating soil microbial communities and nutrient cycling. Plant invasion often increases the availability of soil nutrients, including phosphorus, thus alleviating phosphorus limitation and promoting plant growth [54,55]. Under soil legacy effects, *A*. *philoxeroides* exhibited altered N:P stoichiometry, with a pronounced shift toward greater P investment, reflecting its high plasticity in N and P acquisition and allocation. However, this adjustment in N and P nutrient strategies may impose a cost on *A*. *philoxeroides* during interspecific competition. Although soil legacies enhance its phosphorus acquisition efficiency, the concomitant reduction in nitrogen assimilation constrains its ability to build photosynthetic tissues and synthesize functional proteins. In high-latitude regions, this nitrogen acquisition cost is further exacerbated by declining soil nutrient availability, which may ultimately reduce its invasive dominance in native plant communities [56]. These findings provide a novel perspective for understanding the latitudinal shifts in mechanisms of plant invasion and species coexistence.

### 3.2. Effects of Soil Legacies on Plant Photosynthetic Fluorescence Across Latitudes and Potential Driving Factors

*F*_0_ represents the basal fluorescence when all PSII reaction centers are open after dark adaptation. Its value is positively correlated with chlorophyll concentration and is widely used as an indicator of plant stress status [57]. In this study, relative to the no-legacy controls, soil legacies reduced *F*_0_ in *A. philoxeroides* across all latitudinal clusters. This reduction may be associated with decreased nitrogen content in *A. philoxeroides* under soil legacy effects, which constrained chlorophyll synthesis [58] and consequently reduced electron transport efficiency, thereby lowering *F*_0_ values [57]. *F*_m_ refers to the maximum fluorescence yield when PSII reaction centers are fully closed, reflecting plant physiological performance under specific habitat conditions [59]. In monocultures at low and middle latitudes, soil legacies resulted in significantly higher *F*_0_ and *F*_m_ values for *A. philoxeroides* than for *D. sanguinalis*. However, at higher latitudes, this pattern reversed, with *D. sanguinalis* exhibiting higher values for both indicators. This may be because soils in low- and mid-latitude invaded habitats provide more readily available nutrients and/or promote the accumulation of beneficial growth-promoting microbes, thus enhancing PSII activity and electron transport efficiency in *A*. *philoxeroides* [60]. In contrast, soil properties and microbial community structures in high-latitude invaded habitats seem to constrain the photosynthetic performance of *A. philoxeroides* while simultaneously improving PSII function in *D. sanguinalis*.

Our study revealed a latitudinal reversal in photosynthetic fluorescence parameters between *A*. *philoxeroides* and *D*. *sanguinalis* under soil legacy treatments—specifically, a shift from invasive advantage at low–mid latitudes to native advantage at higher latitudes. These findings are similar to those of Cheng et al. [18], who reported positive soil legacy effects for the invasive *Spartina alterniflora* across latitudes, whereas the legacy effects for its native co-occurring *Phragmites australis* shifted from negative to positive with increasing latitude. This consistency with previous studies suggests that broader driving factors may underlie such asymmetric latitudinal patterns. Latitudinal variation can influence soil nutrient availability by regulating organic matter decomposition, nutrient mineralization, and elemental cycling processes, thereby altering habitat nutrient limitations and modifying plant responses to soil legacies [61,62]. Moreover, plant species with different life-history strategies and resource-use traits may exhibit distinct responses to soil legacies along environmental gradients. Invasive plants generally possess greater phenotypic plasticity and resource acquisition capacity, allowing them to rapidly exploit available resources and maintain growth advantages under changing environments [62,63]. In contrast, native plants that have evolved under local environmental conditions may adopt more conservative resource-use strategies, enabling efficient resource utilization and persistence under nutrient-limited habitats [64]. Together, differences in plant functional types and habitat nutrient limitations jointly shape the asymmetric latitudinal pattern of soil legacy effects between *A. philoxeroides* and *D. sanguinalis* observed in our study.

Compared with the control treatment, soil legacies across all latitudinal clusters eliminated the *F*_m_ advantage of *A*. *philoxeroides* over *D*. *sanguinalis* under mixed culture, likely because of interspecific competition. Plants often alter their resource competition strategies—for light, nutrients, and water—to increase their own growth advantages [65]. Previous studies have shown that invasive plants are susceptible to negative soil legacy effects during long-term soil occupation, as pathogen accumulation and depletion of beneficial microbes may inhibit resource acquisition and photosynthetic activity [31,60,66]. In this study, *A*. *philoxeroides* enhanced its *F*_m_ advantage by improving resource acquisition in a control treatment with relatively scarce soil nutrients. However, it may suffer from strong negative feedback under soil legacy effects with invaded soils, resulting in reduced photosynthetic activity [58]. In contrast, *D*. *sanguinalis* is less susceptible to specific soil pathogens and can fully utilize resources in invaded soils, along with beneficial microbes such as phosphate-solubilizing and nitrogen-fixing bacteria, to maintain high photosynthetic activity [5,67]. Soil legacies at high latitudes resulted in both *F*_0_ and *F*_m_ being higher in *D*. *sanguinalis* than in *A*. *philoxeroides* under monoculture. This is because the lower annual average temperature and reduced resource availability at high latitudes further increase the negative effects of soil legacy on *A*. *philoxeroides* [66]. As a native species, *D*. *sanguinalis* may be more likely to maintain stable and high resource utilization efficiency because of its longer interaction history with native soil microorganisms, thus maintaining high photosynthetic activity under resource-limited conditions [68].

Our study revealed latitude-dependent shifts in the dominant correlates of photosynthetic fluorescence between *A. philoxeroides* and *D. sanguinalis* under soil legacy effects. With respect to low-latitude soil legacies, the photosynthetic fluorescence traits of *D. sanguinalis* showed stronger associations with shoot biomass and total carbon, whereas those of *A. philoxeroides* were more strongly associated with ecological stoichiometry and root length. These findings suggest that resource-rich and microbially active soil environments at low latitudes may promote divergent resource-use strategies between invasive and native plant species [69]. Previous studies have similarly shown that invasive plants often increase carbon assimilation by allocating more nitrogen to photosynthetic organs, whereas native species tend to adopt more conservative resource-use strategies [47,70]. Across latitudes, however, the dominant correlates of photosynthetic fluorescence converged between the two species, with ecological stoichiometry becoming more prominent in the middle- and high-latitude clusters. This convergence indicates that under resource-limited and stressful conditions, photosynthetic performance may depend more heavily on nutrient allocation and elemental balance—a pattern that is consistent with the stoichiometric homeostasis theory [44], which posits that nutrient limitation governs plant physiological processes.

### 3.3. Limitations and Future Perspectives

Several limitations should be considered when interpreting our findings. First, although our previous studies have shown that *A*. *philoxeroides* and *D*. *sanguinalis* are widely distributed across broad latitudinal gradients and exhibit substantial local adaptation [24], and we used species-specific propagation methods to better represent their natural recruitment strategies for pot experiments, the clonal reproduction of *A*. *philoxeroides* versus the seed reproduction of *D*. *sanguinalis* may result in divergent initial resource status and early root development between the two species prior to transplantation, thereby introducing potential initial biases [71]. Second, our experiments do not include a sterilized invaded-soil treatment; the potential influence of abiotic soil properties on the experimental results thus cannot be completely excluded.

Future studies could address these limitations using several methods. First, using additional species pairs with contrasting or comparable propagation methods in multi-latitude population trials would help assess the extent to which initial condition biases influence soil legacy estimates. Second, experiments including paired live versus sterilized aliquots of the same field soil, as well as two-phase plant–soil feedback (PSF) experiments, could help disentangle microbial effects from abiotic soil effects. Finally, integrating soil microbial high-throughput sequencing in future studies would allow the identification of the species composition, diversity levels, and functional attributes of soil microbial communities in different invaded habitats, thereby providing a more comprehensive understanding of the mechanisms underlying plant–soil interactions across latitudes.

## 4. Materials and Methods

### 4.1. Soil Collection

**Figure 7:**
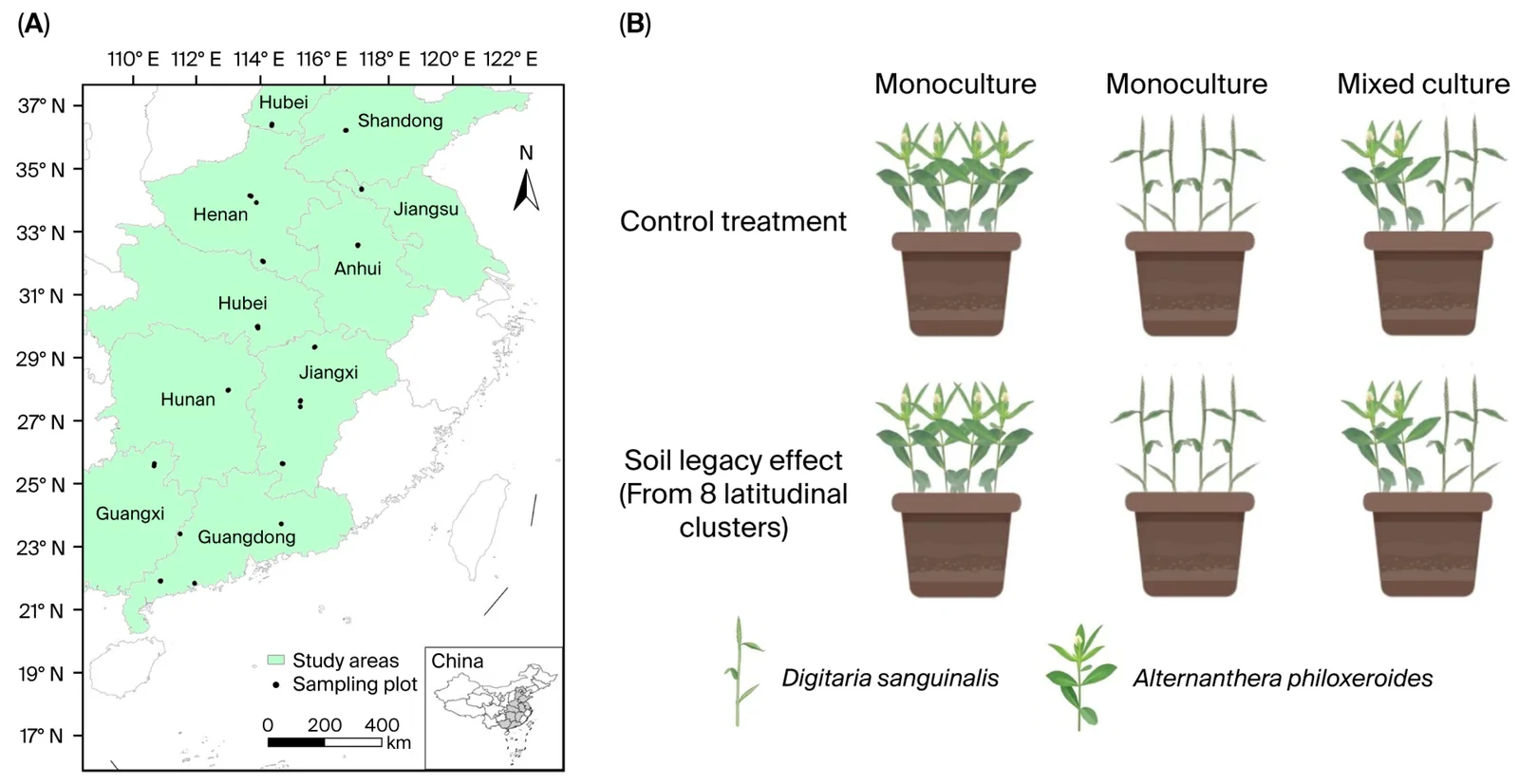
Experimental design of this study. Panel (**A**): The soil collection sites for the field-invaded habitats of *A*. *philoxeroides* spanned eight latitudinal clusters, ranging from 21° N to 37° N. Panel (**B**): Based on the De Wit replacement method, a pot experiment was established in the greenhouse with three factors: soil source, planting type, and species identity.

During the peak growing season from July to August, soil samples were collected from 40 plots located in habitats invaded by terrestrial *A*. *philoxeroides* along a latitudinal gradient (21–37° N) in China, with sampling sites distributed across 10 provinces and 17 cities (Figure 7A). Eight latitudinal clusters were established at 2° intervals: low-latitude clusters (cluster 1: 21–23° N; cluster 2: 23–25° N), middle-latitude clusters (cluster 3: 25–27° N; cluster 4: 27–29° N; cluster 5: 29–31° N; cluster 6: 31–33° N), and higher-latitude clusters (cluster 7: 33–35° N; cluster 8: 35–37° N). In each cluster, five plots were selected in areas where the continuous invasion of *A*. *philoxeroides* exceeded 100 m^2^, and adjacent plots within the same cluster were separated by more than 10 km to ensure spatial independence [24]. A handheld GPS (eTrex 20, Garmin Ltd., Lenexa, KS, USA) was used to record the geographic coordinates and altitude data of the plot habitats. After surface litter and other debris were removed, approximately 200 g of soil from the 0 to 20 cm layer was collected from each plot using the five-point sampling method [72]. The five subsamples from each plot were homogenized to form one composite sample per plot. Roots, animal and plant residues, and gravel were carefully removed by hand. Each composite soil sample was placed in a sterile sealed bag, stored at 4 °C in a portable cooler, and transported to the laboratory within 48 h. For each latitudinal cluster, the five composite samples from its five plots were homogenized, passed through a 2 mm sieve, and pooled. The pooled sample was maintained at 4 °C, and the soil legacy effect experiment was initiated within one week of storage [73].

### 4.2. Plant Materials

The original populations of *A*. *philoxeroides* and *D*. *sanguinalis* seeds used in this study were collected from a naturally co-occurring community of these two species in the wild near Xinyang City (32.450° N, 114.059° E), Henan Province, China. *A*. *philoxeroides* was propagated by cuttings, with 2 cm stem segments containing at least one node cut and buried in seedling trays for germination. *D*. *sanguinalis* was grown from seeds, with plump, healthy, and undamaged seeds sown in seedling trays. All trays were maintained in an artificial climate chamber (MGC-400H, Shanghai Yiheng Technology Instrument Co., Ltd., Shanghai, China) under controlled conditions: 14 h light (25 °C, 70% relative humidity, 600 µmol·m^−2^·s^−1^) and 10 h dark (18 °C, 60% relative humidity). After seedlings had grown two true leaves, individuals of both species with similar growth vigor were selected for the subsequent soil legacy experiment [59].

### 4.3. Experimental Design

The pot experiment employed a three-factor design—soil source (control vs. soil legacies from eight latitudinal clusters), planting type (monoculture vs. mixed culture), and species identity (*D*. *sanguinalis* vs. *A*. *philoxeroides*)—with six replicates per treatment combination (Figure 7B). The five field plots within each cluster were pooled before the greenhouse experiment and treated as subsamples contributing to a pooled soil source rather than as independent experimental replicates. Soil source was treated as a categorical factor with nine levels: one sterilized control and eight pooled invaded-habitat soil sources corresponding to the eight latitudinal clusters described in Section 4.1 (21–37° N at 2° intervals). The substrate, composed of nutrient soil, perlite, and fine sand at a volume ratio of 4:2:1, was sterilized at 121 °C for 40 min in an autoclave (BKQ-B120II, Boko Holdings Group Co., Ltd., Shandong, China) [59]. Each plastic pot (20 cm diameter × 17 cm height) was then filled with 750 g of the sterilized substrate. For the live-soil inoculum treatment, 150 g of field-collected soil was added to each pot and thoroughly mixed with the sterilized substrate. The total substrate mass per pot was 900 g, with field-collected soil accounting for 1/6 of the total mass [74]. In the control treatment, 150 g of sterilized substrate was added instead. Given that *A*. *philoxeroides* invasion enriches soil nitrogen and organic carbon and drives nutrient homogenization [25,75], we omitted a sterilized invaded-soil treatment to isolate the effects of latitudinally varying soil nutrients on legacy effects. A De Wit replacement series was used to maintain a constant total density of four plants per pot: The monoculture consisted of either four *D*. *sanguinalis* or four *A*. *philoxeroides*, while the mixed culture contained two individuals of each species [59]. All pots were maintained in a controlled greenhouse at 25 °C and 60% relative humidity. The plants were watered every two days using a weighing method to maintain the soil moisture at approximately 70% of the field capacity. Pot positions were rerandomized every 10 days to minimize positional effects. After 50 days of cultivation, we measured plant morphology, biomass, tissue nutrient content, and photosynthetic fluorescence.

### 4.4. Indicator Measurement

Photosynthetic fluorescence indicators were measured using a chlorophyll fluorescence imaging system (FluorCam, Photon Systems Instruments, Brno, Czech Republic) after intact leaves were dark-adapted for 15 min. Minimum fluorescence (*F*_0_) was recorded in the dark, followed by a saturating pulse (800 ms, 3000 µmol·m^−2^·s^−1^) from a blue LED (peak wavelength 449 nm) to determine maximum fluorescence (*F*_m_). Variable fluorescence (*F*_v_ = *F*_m_ − *F*_0_) and the maximum photochemical efficiency (*F*_v_/*F*_m_) were subsequently calculated. Under light-adapted steady-state conditions, photochemical quenching (qP_Lss), steady-state fluorescence decay rates (Rfd_Lss), and steady-state actual photochemical efficiencies (QY_Lss) were obtained according to the predefined actinic-light protocol. The maximum stem length (MSL) per plant was measured with a steel ruler. Leaves and roots were subsequently washed and blotted dry, after which the leaf area (LA), root area (RA), root length (RL), root volume (RV), and root diameter (RD) were measured using a ScanMaker i800 scanning system (Microtek, China). All plants in each pot were harvested, washed, and dried in a forced-air oven (101-4A, Shanghai Leiyunshiyan Instrument Manufacturing Co., Ltd., Shanghai, China) at 75 °C for 48 h to a constant weight. The total biomass (TB), shoot biomass (SB), and root biomass (RB) were weighed using an analytical balance (0.0001 g precision; FA224, Shanghai Lichen Instrument Technology Co., Ltd., Shanghai, China), and the root–shoot ratio (RSR) was calculated accordingly. The dried plant material was ground and passed through a 70-mesh sieve. In total, ~4 mg of plant powder in tin capsules was analyzed for the total carbon (TC) and total nitrogen (TN) using a Vario TOC Select analyzer (Vario TOC, Elementar Analysensysteme GmbH, Langenselbold, Germany) via high-temperature dry combustion at 950 °C under oxygen-assisted catalytic conditions [76], while blank samples and certified reference materials were included for calibration and quality control [77]. Moreover, ~0.20 g of plant powder was digested with H_2_SO_4_–H_2_O_2_ at 360 °C until the digest became clear. Total phosphorus (P) concentrations were then determined via the molybdenum blue method using a continuous flow analyzer (Futura, AMS Alliance, Paris, France) [78]. Ecological stoichiometric ratios (C:N, C:P, and N:P) were subsequently calculated [8,59].

### 4.5. Statistical Analyses

We conducted multi-way ANOVA using SPSS 16.0 software (SPSS Inc., Chicago, IL, USA) to examine the effects of soil treatment, planting type, species attribute, and their interactions on the morphology, biomass, nutrient content, and photosynthetic fluorescence of *D*. *sanguinalis* and *A. philoxeroides*. Prior to ANOVA, all data were tested for homogeneity of variance. If the data were not homogeneous, square-root or logarithmic transformation was applied to meet this assumption. For treatment combinations with significant ANOVA results (*p* < 0.05), the least significant difference (LSD) method was used for multiple comparisons to determine the source of variability. We preferentially analyzed the results with significant interaction effects involving soil source and other factors, as well as results showing a significant single effect of soil source.

To examine the relationships between plant growth and photosynthetic fluorescence at the whole-plant level, we performed partial redundancy analysis (pRDA) using the rda() function in the vegan package (R 4.5.1, R Foundation for Statistical Computing, Vienna, Austria) [79], with *F*_0_, *F*_m_, *F*ᵥ/*F*_m_, QY_Lss, qP_Lss, and Rfd_Lss as response variables; latitude as a covariate; and plant morphological, biomass, and nutrient traits as explanatory variables. By conditioning on latitude (Condition(latitude)), we assessed the associations between explanatory variables and fluorescence variation after removing latitudinal effects. The significance of each explanatory variable was tested using 999 Monte Carlo permutations, and the adjusted *R*^2^ of the global model was reported [80]. To further validate these results on the full dataset and identify dominant factors within different latitudinal clusters, we performed separate redundancy analysis (RDA) for each cluster using Canoco software (Canoco 4.5, Microcomputer Power, Ithaca, NY, USA)), with dominant factors identified via forward selection based on permutation tests [72].

## 5. Conclusions

We found that soil legacies originating from invaded habitats across different latitudinal clusters differentially affected the growth and photosynthetic performance of invasive *A*. *philoxeroides* and native *D*. *sanguinalis*. Low-latitude soil legacies generally showed stronger growth-promoting effects on *D*. *sanguinalis*, whereas *A*. *philoxeroides* showed no significant morphological responses to soil legacies. In contrast, middle- and high-latitude soil legacies were more closely associated with variations in ecological stoichiometric traits. Meanwhile, the variables most strongly associated with photosynthetic fluorescence shifted from growth-related traits in low-latitude clusters toward ecological stoichiometric traits in middle- and high-latitude clusters. Notably, soil legacies eliminated the photosynthetic advantages of *A*. *philoxeroides* over *D*. *sanguinalis* in mixed culture. These findings provide new insights for latitude-specific management; however, they are applicable only to the system of coexisting terrestrial *A*. *philoxeroides* and *D*. *sanguinalis*. At low latitudes, the substantial growth advantages of *D*. *sanguinalis* under soil legacies suggest that it may serve as a promising candidate for replacement control, although field validation is needed. In middle- and high-latitude clusters, management should instead focus on reducing soil phosphorus availability (e.g., via P-fixing amendments) to intensify phosphorus limitation and thereby suppress the spread of *A*. *philoxeroides*. Our study generates hypotheses concerning how soil legacies originating from different latitudes may influence invasive–native interactions.

## Figures and Tables

**Table 1:** Three-way ANOVA for the effects of soil source (S), planting type (P), species identity (I) and their interactions on plant morphology.

Source	*df*	Maximum Stem Length		Leaf Area		Root Length		Root Area		Root Volume		Root Diameter	
		*F*	*p*	*F*	*p*	*F*	*p*	*F*	*p*	*F*	*p*	*F*	*p*
S	8, 144	6.084	<0.001	4.735	<0.001	1.019	0.425	3.309	0.002	1.287	0.255	4.085	<0.001
P	1, 144	0.962	0.328	0.521	0.472	4.300	0.040	17.727	<0.001	12.513	0.001	52.439	<0.001
I	1, 144	751.604	<0.001	655.002	<0.001	31.496	<0.001	8.874	0.003	0.921	0.339	43.545	<0.001
S × P	8, 144	0.761	0.638	1.127	0.349	1.983	0.053	1.992	0.051	1.382	0.209	1.976	0.053
S × I	8, 144	2.931	0.005	3.294	0.002	0.551	0.817	0.823	0.584	1.366	0.216	1.207	0.299
P × I	1, 144	0.254	0.615	1.440	0.232	0.437	0.509	1.914	0.169	0.273	0.602	6.190	0.014
S × P × I	8, 144	1.129	0.347	1.443	0.184	0.828	0.580	1.159	0.328	1.199	0.303	1.256	0.271

**Table 2:** Three-way ANOVA for the effects of soil source (S), planting type (P), species identity (I) and their interactions on plant biomass.

Source	*df*	Total Biomass		Shoot Biomass		Root Biomass		Root–Shoot Ratio	
		*F*	*p*	*F*	*p*	*F*	*p*	*F*	*p*
S	8, 144	2.686	0.009	3.904	<0.001	1.036	0.412	2.215	0.030
P	1, 144	43.185	<0.001	34.842	<0.001	35.885	<0.001	3.002	0.085
I	1, 144	29.319	<0.001	6.981	0.009	180.461	<0.001	456.653	<0.001
S × P	8, 144	1.535	0.150	1.025	0.420	1.493	0.165	0.716	0.677
S × I	8, 144	0.878	0.537	1.455	0.179	0.496	0.857	0.465	0.879
P × I	1, 144	0.526	0.469	3.900	0.050	14.700	<0.001	12.927	<0.001
S × P × I	8, 144	0.829	0.578	0.926	0.497	1.003	0.436	0.590	0.785

**Table 3:** Three-way ANOVA for the effects of soil source (S), planting type (P), species identity (I) and their interactions on plant nutrient content.

Source	*df*	Total Carbon (C)		Total Nitrogen (N)		Total Phosphorus (P)		C:N		C:P		N:P	
		*F*	*p*	*F*	*p*	*F*	*p*	*F*	*p*	*F*	*p*	*F*	*p*
S	8, 144	6.671	<0.001	7.240	<0.001	6.693	<0.001	22.115	<0.001	5.094	<0.001	23.671	<0.001
P	1, 144	511.213	<0.001	289.178	<0.001	6.540	0.012	358.752	<0.001	544.086	<0.001	245.738	<0.001
I	1, 144	21.010	<0.001	14.615	<0.001	223.268	<0.001	1.675	0.198	6.548	0.012	30.209	<0.001
S × P	8, 144	7.708	<0.001	2.678	0.009	0.711	0.682	5.767	<0.001	7.720	<0.001	1.578	0.138
S × I	8, 144	1.264	0.268	2.878	0.006	2.803	0.007	0.279	0.972	0.699	0.692	8.451	<0.001
P × I	1, 144	4.975	0.027	3.882	0.051	0.008	0.928	9.262	0.003	6.017	0.016	23.170	<0.001
S × P × I	8, 144	0.887	0.530	0.919	0.504	0.985	0.451	0.582	0.791	0.235	0.984	0.690	0.700

**Table 4:** Three-way ANOVA for the effects of soil source (S), planting type (P), species identity (I) and their interactions on plant photosynthetic fluorescence.

Source	*df*	*F*_0_		*F*_m_		*F*_v_/*F*_m_		qP_Lss		Rfd_Lss		QY_Lss	
		*F*	*p*	*F*	*p*	*F*	*p*	*F*	*p*	*F*	*p*	*F*	*p*
S	8, 144	1.771	0.087	0.750	0.647	0.758	0.640	3.338	0.002	0.293	0.967	1.178	0.316
P	1, 144	0.163	0.687	0.005	0.945	0.154	0.695	0.654	0.420	1.997	0.160	0.755	0.386
I	1, 144	85.001	<0.001	53.760	<0.001	6.793	0.010	27.842	<0.001	0.001	0.978	0.153	0.696
S × P	8, 144	1.623	0.123	0.625	0.756	0.694	0.697	0.487	0.864	1.096	0.370	1.134	0.344
S × I	8, 144	5.145	<0.001	3.412	0.001	1.550	0.145	1.215	0.294	0.350	0.945	0.530	0.832
P × I	1, 144	4.060	0.046	4.365	0.038	0.194	0.660	2.100	0.149	0.763	0.384	0.219	0.641
S × P × I	8, 144	4.329	<0.001	2.846	0.006	0.817	0.589	1.703	0.102	0.271	0.974	0.555	0.813

## Data Availability

Data will be made available upon request.

## Supplementary Materials

The following supporting information can be downloaded at: https://www.mdpi.com/article/10.3390/plants15182792/s1, Table S1. Significance tests of the contributions of explanatory variables in the partial redundancy analysis (pRDA) model; Table S2. Correlations of plant growth indicators with the first two axes of the redundancy analysis (RDA).
