# Supplementary material for: Latitudinal Variation in the Responses of Invasive Alternanthera philoxeroides and Native Digitaria sanguinalis to Soil Legacy Effects
Source: Plants (Basel). 2026 Sep 11;15(18):2792. doi: 10.3390/plants15182792 (PMC13610945; doi:10.3390/plants15182792)
Supplement: Supplementary file 1 [file plants-15-02792-s001.zip › plants-4512423-supplementary.pdf]

Table S1. Significance tests of the contributions of explanatory variables in the partial redundancy analysis (pRDA) model.

Plant Growth Indicators	Variance	Contribution Percent	*F*-value	*P*-value	R^2	Adjusted R^2
TP	0.439	27.73	16.238	0.001	0.082	0.078
LA	0.310	19.62	11.490	0.001	0.120	0.116
TN	0.186	11.78	6.898	0.001	0.013	0.008
MSL	0.111	6.99	4.097	0.013	0.126	0.122
CP	0.090	5.71	3.345	0.021	0.003	-0.003
TC	0.078	4.93	2.888	0.034	0.013	0.008
RL	0.069	4.37	2.558	0.042	0.025	0.020
NP	0.056	3.55	2.082	0.085	0.024	0.019
RV	0.060	3.81	2.229	0.048	0.009	0.004
RA	0.052	3.26	1.906	0.092	0.021	0.016
RD	0.050	3.15	1.843	0.111	0.025	0.020
TB	0.025	1.56	0.915	0.426	0.032	0.027
RSR	0.020	1.27	0.744	0.523	0.116	0.112
CN	0.015	0.92	0.540	0.656	0.018	0.013
SB	0.013	0.81	0.477	0.728	0.027	0.022
RB	0.009	0.54	0.315	0.806	0.076	0.071

Definition of abbreviations: TP—total phosphorus; LA—leaf area; TN—total nitrogen; MSL—maximum stem length; CP—carbon–phosphorus ratio (C:P); TC—total carbon; RL—root length; NP—nitrogen–phosphorus ratio (N:P); RV—root volume; RA—root area; RD—root diameter; TB—total biomass; RSR—root–shoot ratio; CN—carbon–nitrogen ratio (C:N); SB—shoot biomass; and RB—root biomass.

Table S2. Correlations of plant growth indicators with the first two axes of the redundancy analysis (RDA).

Plant Growth Indicators	Low-Latitude Soil Legacies				Middle-Latitude Soil Legacies				High-Latitude Soil Legacies			
	D. sanguinalis		*A. philoxeroides*		*D. sanguinalis*		*A. philoxeroides*		*D. sanguinalis*		*A. philoxeroides*	
	Axis 1	Axis 2	Axis 1	Axis 2	Axis 1	Axis 2	Axis 1	Axis 2	Axis 1	Axis 2	Axis 1	Axis 2
MSL	0.698	0.509	0.364	0.881*	0.441	0.540	0.900**	0.154	0.944*	-0.104	0.924*	0.118
LA	-0.189	0.739	0.267	0.277	-0.110	0.576	0.440	-0.007	0.601	-0.455	0.772	-0.510
RA	0.882*	0.450	0.908*	0.386	0.650	0.238	0.904**	0.207	-0.107	-0.154	0.806	-0.056
RD	0.911*	0.393	0.856	0.516	0.776*	0.038	0.889**	-0.225	-0.838	-0.155	0.984**	-0.052
RL	0.785	0.535	0.920*	0.325	0.575	0.424	0.687*	0.305	-0.635	-0.366	-0.836	0.508
RV	0.946*	0.320	0.824	0.563	0.618	0.106	0.731*	0.245	-0.736	-0.418	0.265	-0.015
TB	-0.899*	0.297	-0.380	0.916	-0.672*	0.658	-0.694*	0.427	0.793	-0.180	0.917*	0.114
SB	-0.986**	0.055	-0.470	0.864	-0.716*	0.627	-0.532	0.330	0.821	-0.156	-0.864	0.481
RB	-0.954*	0.258	-0.273	0.959**	-0.460	0.754*	-0.768*	0.435	0.696	-0.302	-0.778	0.543
RSR	0.740	0.655	0.076	-0.938*	0.731*	-0.070	-0.845**	0.055	-0.778	-0.596	0.234	0.969**
TC	0.965**	0.258	0.853	-0.288	0.936**	-0.212	0.959**	-0.041	-0.999**	0.007	0.930*	-0.280
TN	0.923*	0.319	0.826	-0.270	0.893**	-0.324	0.930**	-0.095	-0.999**	0.033	0.962**	-0.215
TP	0.364	-0.478	-0.443	-0.121	0.087	-0.445	0.724*	-0.122	-0.772	0.600	0.874	-0.230
CN	0.942*	0.322	0.904*	-0.271	0.944**	-0.139	0.938**	-0.001	-0.996**	0.034	0.870	-0.345
CP	0.857	0.483	0.922*	-0.202	0.928**	-0.161	0.969**	-0.054	-0.992**	-0.101	0.963**	-0.228
NP	0.725	0.659	0.912*	-0.191	0.893**	-0.207	0.950**	-0.113	-0.993**	-0.114	0.964**	-0.225

$^{*}P < 0.05$; $^{**}P < 0.01$.

Definition of abbreviations: MSL—maximum stem length; LA—leaf area; RA—root area; RD—root diameter; RL—root length; RV—root volume; TB—total biomass; SB—shoot biomass; RB—root biomass; RSR—root–shoot ratio; TC—total carbon; TN—total nitrogen; TP—total phosphorus; CN—carbon–nitrogen ratio (C:N); CP—carbon–phosphorus ratio (C:P); and NP—nitrogen–phosphorus ratio (N:P).
